# Personality’s voice: how personality traits shape verbal processes in collaborative problem-solving

**DOI:** 10.3389/fpsyg.2026.1830668

**Published:** 2026-07-22

**Authors:** Siem Buseyne, Fien Depaepe, Annelies Raes

**Affiliations:** 1 Department of Training and Education Sciences, Faculty of Social Sciences, University of Antwerp, Antwerpen, Belgium; 2 KU Leuven, Faculty of Psychology and Educational Sciences, Centre for Instructional Psychology and Technology, Leuven, Belgium; 3 Centre Interuniversitaire de Recherche en Education de Lille, Université de Lille, Villeneuve d’Ascq, France; 4 KU Leuven, imec research group itec, Leuven, Belgium

**Keywords:** collaborative problem solving, linguistic inquiry and word count, personality, teamwork, verbal interaction

## Abstract

This exploratory study examined the associations between personality traits and verbal interactions during collaborative problem solving (CPS) in adult workplace teams. Using the Business Attitudes Questionnaire, we examined personality domains, facets, and compounds in relation to CPS-specific verbal behaviors and linguistic indicators. Data were collected from 36 participants across nine professional teams during a CPS training exercise consisting of multiple problem-solving intervals. Verbal interactions were analyzed through quantitative content analysis, in which utterance-level CPS behaviors were coded and transformed into relative frequencies, complemented by dictionary-based linguistic analysis using Linguistic Inquiry and Word Count. The analyses were treated as exploratory. Multilevel models accounting for repeated observations within individuals suggested that Openness, particularly the Change-Oriented facet, was linked to greater knowledge sharing and cognitive process word use. Ambitious individuals tended to contribute more to shared knowledge but less to coordination and negotiation, while Results-oriented individuals tended to show higher word counts, and Strategic individuals tended to use more analytical language. The study serves as an initial empirical mapping of possible theory-informed links between personality and verbal CPS processes and identifies directions for future confirmatory research.

## Introduction

Collaborative problem solving (CPS) refers to a process in which multiple individuals work together to solve a problem by exchanging knowledge, skills, and resources in pursuit of a shared solution (OECD, 2017). Recent technological advancements have substantially expanded the ways in which these collaborative processes can be supported, studied, and analyzed (e.g., Ortega, 2021). In particular, developments in artificial intelligence and natural language processing (NLP) have enabled more advanced and increasingly (semi-)automated forms of data processing, such as facial emotion recognition and automatic speech recognition. These developments have stimulated growing interest in the analysis of both verbal and non-verbal indicators of collaboration (e.g., Praharaj et al., 2023). At the same time, research has increasingly examined factors that may shape team behavior in collaborative environments (e.g., Hsu et al., 2008). These factors include individual characteristics of team members, such as gender, ethnicity, age, and personality traits; group characteristics, such as team composition; task characteristics; and technology use. According to Graesser et al. (2018), social elements play a pivotal role in the success of CPS processes. One factor that has received growing attention is personality. For example, studies have examined how team composition based on personality traits is associated with group performance outcomes (Liang et al., 2015). Similarly, in organizational psychology, increasing attention has been paid to the links between personality and team performance, as well as other important life outcomes (Stanek and Ones, 2023). Despite these developments, the role of personality in CPS remains insufficiently understood (Graesser et al., 2018). In particular, research on the relationship between team members’ personality traits and CPS behaviors is still limited (Jolić Marjanović et al., 2024). The present paper addresses this gap by investigating verbal interactions during adult CPS using multiple analytic techniques.

## Theoretical framework

CPS can be understood as a dynamic system in which multiple factors interact to shape how teams collaborate and perform (Graesser et al., 2018). A useful framework for structuring this system is the Context–Input–Mediator–Output–Input (CIMOI) framework, which conceptualizes teamwork as a cyclical process in which contextual conditions and team inputs influence team mediators, which in turn shape team outputs and may feed back into future collaboration (Ilgen et al., 2005; Scott and Wildman, 2017). Compared with earlier Input–Process–Output models (e.g., Scheerens, 2015), the CIMOI framework places greater emphasis on the broader context of collaboration, the dynamic nature of team functioning, and the role of mediating mechanisms through which inputs are translated into outcomes.

Applied to CPS, the CIMOI framework emphasizes that team performance arises from the interplay between what team members bring into the collaboration and how they interact during the problem-solving process. Team inputs may include relatively stable individual characteristics, such as personality or team roles (Buseyne et al., 2024b; Mathieu et al., 2015), whereas team mediators refer to the mechanisms through which collaboration unfolds. These team mediators are often divided into team processes and emergent states (Scott and Wildman, 2017). Team processes refer to the observable actions and interactions. Emergent states, in contrast, refer to more implicit and dynamic team properties, such as trust, cohesion, and team flow (Buseyne et al., 2025; van den Hout et al., 2024, 2026). In CPS, communication is a particularly important team process. Verbal interaction provides one of the most direct ways to examine how team members construct shared understanding, negotiate, coordinate, and maintain team functioning (Buseyne et al., 2024a; Graesser et al., 2018).

The present study focuses on two components within the CIMOI framework. First, we examine verbal interaction in CPS as a key team process within the broader category of team mediators, as many CPS processes are enacted through communication. Second, we consider personality as an individual input factor, given that personality differences may shape how individuals approach and contribute to collaboration. We then review prior research linking personality to verbal interaction.

### Verbal interaction in CPS

Many CPS mechanisms require complex verbal interactions among team members (Cukurova et al., 2018). To study such interactions among learners, various frameworks and methods can be used, which are both qualitative and quantitative of nature (Buseyne et al., 2024a). These methods mostly rely on video, consisting of both visual and acoustic data (Knoblauch et al., 2014). A research method often used to unravel the meaning making process of dialogues between team members using these data types is content analysis (Jeong et al., 2014). In content analysis, parts of transcriptions are assigned to certain defined categories, enabling data reduction and subsequent quantitative analysis to describe the relative frequency of the categories or the total duration of certain categories (Cohen et al., 2007).

In the context of CPS, a framework and corresponding coding scheme often used in research (e.g., Stewart et al., 2019, 2023) is the one of Sun et al. (2020). This framework consists of three main CPS categories: constructing shared knowledge, negotiation and coordination, and maintaining team function. The first category, (a) constructing shared knowledge, involves sharing and integrating ideas to build a common understanding, supported by behaviors like proposing solutions and clarifying misunderstandings; (b) negotiation and coordination emphasize reaching agreement on plans and monitoring execution, with key actions including providing rationales and aligning efforts; and (c) maintaining team function focuses on sustaining positive dynamics through role fulfillment, encouragement, and minimizing distractions. In the corresponding coding scheme (Sun et al., 2020, 2022), each category includes sub-categories and specific indicators that provide a comprehensive approach to assessing CPS processes.

Aside from examining verbal interactions at the utterance level, as described above, alternative NLP approaches for word-level analysis of verbal interactions are available, such as Linguistic Inquiry and Word Count (LIWC; Pennebaker et al., 2015b,^2022^). LIWC is a software tool designed to analyze both written and spoken language, categorizing it based on a predefined dictionary comprising various categories. These categories encompass linguistic elements like pronouns, prepositions, and articles, as well as psychological attributes, including emotions and cognitive processes, in addition to summary statistics such as word count and words per sentence (e.g., Koutsoumpis et al., 2022; Tausczik and Pennebaker, 2010). Various LIWC categories are relevant in the analysis of CPS interactions (e.g., Buseyne et al., 2024a). For instance, word count and words per sentence serve to quantify individual participation within a team and personal pronouns, including both inclusive (e.g., we, us) and self-oriented personal pronouns (e.g., I), contribute to group membership and cohesion (Demmans Epp et al., 2017). Personal pronouns also reflect one’s orientation and status within the group. Specifically, higher-status individuals tend to involve others, while lower-status members exhibit more self-oriented language using first-person pronouns (Tausczik and Pennebaker, 2010). On the other hand, negations (e.g., no, never) and interrogatives (e.g., how, what) are linked to specific behaviors. Whereas negations can be used to express opposition during negotiation processes (Stewart et al., 2019), interrogatives relate to questioning actions, like seeking clarification or challenging team members’ solutions. Furthermore, emotions, both positive and negative, can provide insights into cognitive, motivational, and relational aspects of CPS (Avry et al., 2020; Avry and Molinari, 2021) and are, according to Tausczik and Pennebaker (2010) effectively detected by positive (e.g., nice) and negative (e.g., hate) emotion words from LIWC. In addition, certain cognitive process words (e.g., think, know, perhaps) correlate with negotiation and coordination (Stewart et al., 2019) and time-related words (e.g., end, until) relate to time management, reflecting how team members handle time constraints (Meier et al., 2007).

### Personality as an individual input in CPS

Team members may differ substantially in how they participate in interaction processes. To better understand such variation, it is important to consider individual input factors that may shape collaborative behavior. One such factor is personality. Personality psychology focuses on understanding human nature by exploring individual differences and consistent behavioral patterns. Central to this field are personality traits, which are enduring behavioral patterns that aid in comprehending an individual’s consistent actions over time (Barenbaum and Winter, 2008).

Various models categorize personality traits, with the Big Five (McCrae and Costa, 2008a,2008b) being the most widely used and accepted framework for understanding personality traits among scientists. This model includes the domains Openness, Conscientiousness, Extraversion, Agreeableness, and Neuroticism. It is worth noting that Agreeableness is sometimes referred to as Altruism, while Neuroticism is often described as the opposite of Positive Emotionality.

Openness captures an individual’s inclination toward embracing new ideas, creativity, and exploring unfamiliar opportunities. People with high levels of Openness are often imaginative, curious, and receptive to change and unconventional perspectives (DeYoung et al., 2010; McCrae and Costa, 1983). In contrast, Conscientiousness is marked by traits such as self-discipline, organization, and a strong sense of responsibility (Peeters et al., 2006). Extraversion highlights sociability, assertiveness, and a preference for external stimulation. Agreeableness emphasizes the degree to which individuals demonstrate kindness, cooperation, and compassion in their interactions (Barrick et al., 1998). Lastly, Neuroticism describes a tendency toward experiencing negative emotions such as anxiety, worry, and sadness, with higher Neuroticism scores indicating greater emotional instability (Peeters et al., 2006).

Each of the personality domains, also referred to as factors, includes various aspects (e.g., Stanek and Ones, 2018). For example, Extraversion consists of the aspects Assertiveness and Enthusiasm (DeYoung et al., 2007). A subsequent lower level in the personality hierarchy consists of personality facets, which are composed of responses to various items in a personality questionnaire (Stanek and Ones, 2018).

Apart from the general personality questionnaires (e.g., the Big Five Inventory; Soto and John, 2017), there are specific contextualized personality models and instruments tailored for various environments, such as workplaces and collaborative settings (Burch and Anderson, 2008). One example is the Business Attitudes Questionnaire (BAQ; Bogaert et al., 2014; Vrijdags et al., 2014; Wille et al., 2018). Table 1 provides an overview of the BAQ domains and their associated facets. In addition, the BAQ also includes five compound facets grouped under Professionalism: Ambitious, Critical, Results-oriented, Strategic, and Autonomous. These compounds are personality attributes that cannot be adequately explained by a single overarching trait, such as one of the Big Five domains (Stanek and Ones, 2018, 2023). Among these compounds, (a) Ambitious denotes a determined effort to establish and accomplish goals; (b) Critical represents a thorough and analytical approach to evaluating information and ideas, often emphasizing the identification of limitations or flaws; (c) Results-oriented highlights a strong focus on achieving significant accomplishments, fueled by a competitive mindset; (d) Strategic refers to a proactive and forward-thinking perspective, prioritizing both immediate and future objectives; and (e) Autonomous reflects the ability to shape one’s environment, adapt to changing situations, and assert individual perspectives, preferences, and unique methods (Bogaert et al., 2014; Vrijdags et al., 2014; Wille et al., 2018). In the context of this study, the BAQ conceptualization is further adopted because of its alignment with workplace and collaborative settings.

**TABLE 1:** Overview of the BAQ domains and facets.

Emotional stability	Extraversion	Openness	Altruism	Conscientiousness
(1) Relaxed	(1) Leading	(1) Abstract	(1) People-oriented	(1) Organized
(2) Optimistic	(2) Communicative	(2) Innovative	(2) Cooperating	(2) Meticulous
(3) Stress-resistant	(3) Persuasive	(3) Change-oriented	(3) Helpful	(3) Rational
(4) Decisive	(4) Motivating	(4) Open-minded	(4) Socially Confident	(4) Persevering

### Links between personality and verbal interaction

Taken together, personality may be understood as an important individual input factor in CPS. The key question is how such individual differences may be expressed in collaborative behavior, and particularly in verbal interaction. This section therefore reviews prior research linking personality to CPS processes and linguistic indicators.

Jolić Marjanović et al. (2024) synthesized research on the relationship between the Big Five personality domains and CPS. Their work explored how personality at both the individual and team levels influence CPS processes and outcomes. While many studies reviewed by Jolić Marjanović et al. (2024) emphasized performance outcomes, some focused specifically on CPS-related behaviors or processes. Specifically, Curşeu et al. (2019) examined the link between personality and contributions to teamwork, Lin et al. (2015) investigated knowledge-sharing behavior, Stewart et al. (2005) explored associations with social and task roles, and Tasa et al. (2011) studied the relationship with interpersonal behaviors. Jolić Marjanović et al. (2024) also highlighted a notable gap in the literature: only a small proportion of studies addressed individual personality in CPS within professional contexts. Importantly, none of the identified studies examined the relationship between individual personality and CPS processes in adult teams.

Nonetheless, the systematic review by Jolić Marjanović et al. (2024) and the studies included serve as an important basis for the current study. Specifically, the authors found that each of the Big Five personality domains has been associated with individual performance in CPS in at least one study. However, they concluded that Openness and Emotional Stability are generally unrelated to CPS processes. They also suggested that Conscientiousness is likely a crucial individual resource for long-term CPS, though it plays a less significant role in short-term scenarios. Nevertheless, Lin et al. (2015) found that Openness, along with Extraversion, Conscientiousness, and Agreeableness, positively predict knowledge-sharing behaviors. Supporting this, several studies, including Curşeu et al. (2019), have underscored the importance of Extraversion, Agreeableness, and Conscientiousness for teamwork, citing their consistent associations with both task-oriented contributions and positive interpersonal behaviors. Notably, extraverted individuals exert significant influence in CPS by forming dense relationship networks, allowing them to proactively voice ideas, take charge, and exert upward influence (Jolić Marjanović et al., 2024). Additionally, Agreeableness has been shown to play a central role in teamwork behaviors, while Conscientiousness predominantly predicts task-specific aspects of CPS (Tasa et al., 2011).

One of the recommendations made by Jolić Marjanović et al. (2024) is that further diversification in capturing CPS performance and processes is recommended. One promising avenue in this regard is the analysis of linguistic indicators, which can provide a more fine-grained view of how individual differences may be reflected in interaction. Although LIWC has been used in various studies to identify linguistic markers of the Big Five traits, this approach has, to our knowledge, not yet been applied to verbal interactions in CPS contexts. Existing LIWC research has shown small but significant correlations between traits and specific linguistic categories. A meta-analysis of Koutsoumpis et al. (2022) shows that self-reported Emotional Stability, Agreeableness, and Conscientiousness correlated with less frequent use of negative emotion words and self-reported Openness correlated with a higher frequency of larger words. Furthermore, individuals who were rated by others as higher in Emotional Stability used more frequently first-person singular pronouns. In addition, persons rated higher in Extraversion and Openness had a higher word count, and those considered higher in Conscientiousness used more cognitive process words.

Contextual factors play a crucial role in moderating these relationships. In this regard, Trait Activation Theory suggests that traits are more likely to be expressed in environments that provide cues for activating trait-relevant behaviors (Tett et al., 2021). For instance, while studies have shown that Extraversion is positively associated with positive emotion words, this link is stronger in asynchronous tasks, such as written communication, compared to synchronous contexts (Koutsoumpis et al., 2022). Furthermore, while most prior research has focused on written text, such as essays and social media posts, spoken language remains an underexplored domain. Moreover, the meta-analysis by Koutsoumpis et al. (2022) did not include studies explicitly linking personality traits to verbal interactions in collaborative settings. Nevertheless, their findings provide a foundation for developing hypotheses related to CPS, as elaborated on in the following section.

## Research aims and questions

Researchers across disciplines emphasized the necessity for further investigating the interplay between team members’ personality and their behavior within CPS settings (Curşeu et al., 2019; Graesser et al., 2018; Jolić Marjanović et al., 2024). Such a detailed examination of personality is vital for understanding individual differences in CPS contexts and to more effectively tailor interventions and support mechanisms to enhance collaborative (learning) processes and outcomes. However, a significant gap persists in research involving professionals and there is a need to diversify the methods used to measure CPS processes (Jolić Marjanović et al., 2024). Alternative approaches to analyzing verbal interactions include (a) employing content analysis to measure the relative frequencies of CPS categories in utterances using established frameworks, and (b) utilizing LIWC. While numerous studies have examined the relationship between personality and LIWC categories, research involving verbal data sources, instead of written text, remains scarce (Koutsoumpis et al., 2022). To our knowledge, no prior work has specifically investigated the link between personality and LIWC in the context of verbal communication within CPS or other collaborative settings. In addition, previous research has predominantly focused on the Big Five personality domains, overlooking personality facets or compounds, which may serve as stronger predictors of CPS behavior.

Building on these considerations, our study aims to provide an initial exploratory examination of the relationship between personality domains, facets, and compounds on the one hand, and verbal communication on the other hand, within the context of CPS in a professional setting. To achieve this, we employ both content analysis and LIWC to analyze verbal CPS behavior. Three exploratory research questions (RQs) are addressed:

*RQ1*: Which associations can be observed between personality domains (i.e., Emotional Stability, Extraversion, Openness, Altruism, and Conscientiousness) and verbal CPS behavior?

*RQ2*: Which associations can be observed between personality facets and verbal CPS behavior?

*RQ3*: Which associations can be observed between BAQ personality compounds (i.e., Ambitious, Critical, Results-oriented, Strategic, and Autonomous) and verbal CPS behavior?

Based on prior work, several theory-informed expectations were derived to guide the exploratory analyses. As presented in the theoretical framework, at the domain level, prior research suggested that Emotional Stability may show limited or inconsistent associations with CPS-specific verbal behaviors, whereas Openness, Extraversion, Conscientiousness, and Altruism may be positively associated with knowledge-sharing behaviors. Extraversion was also expected to be potentially relevant for coordinating behaviors during CPS. For LIWC indicators, previous research suggested that Emotional Stability, Altruism, and Conscientiousness may be negatively associated with negative emotion words; that Emotional Stability may be positively associated with first-person singular pronouns; that Extraversion and Openness may be positively associated with word count; and that Openness and Conscientiousness may be positively associated with cognitive process words.

In addition to these domain-level expectations, the facet- and compound-level analyses were treated as exploratory extensions. These analyses were included to examine whether more specific personality constructs might reveal tentative patterns that are not visible at the broader domain level.

## Materials and methods

### Ethical considerations

This study was approved by the Data Protection Officer and the Ethical Committee of KU Leuven (G-2022-5202). Before taking part, all participants received a letter explaining the study and signed a consent form.

### Research design

**FIGURE 1:**
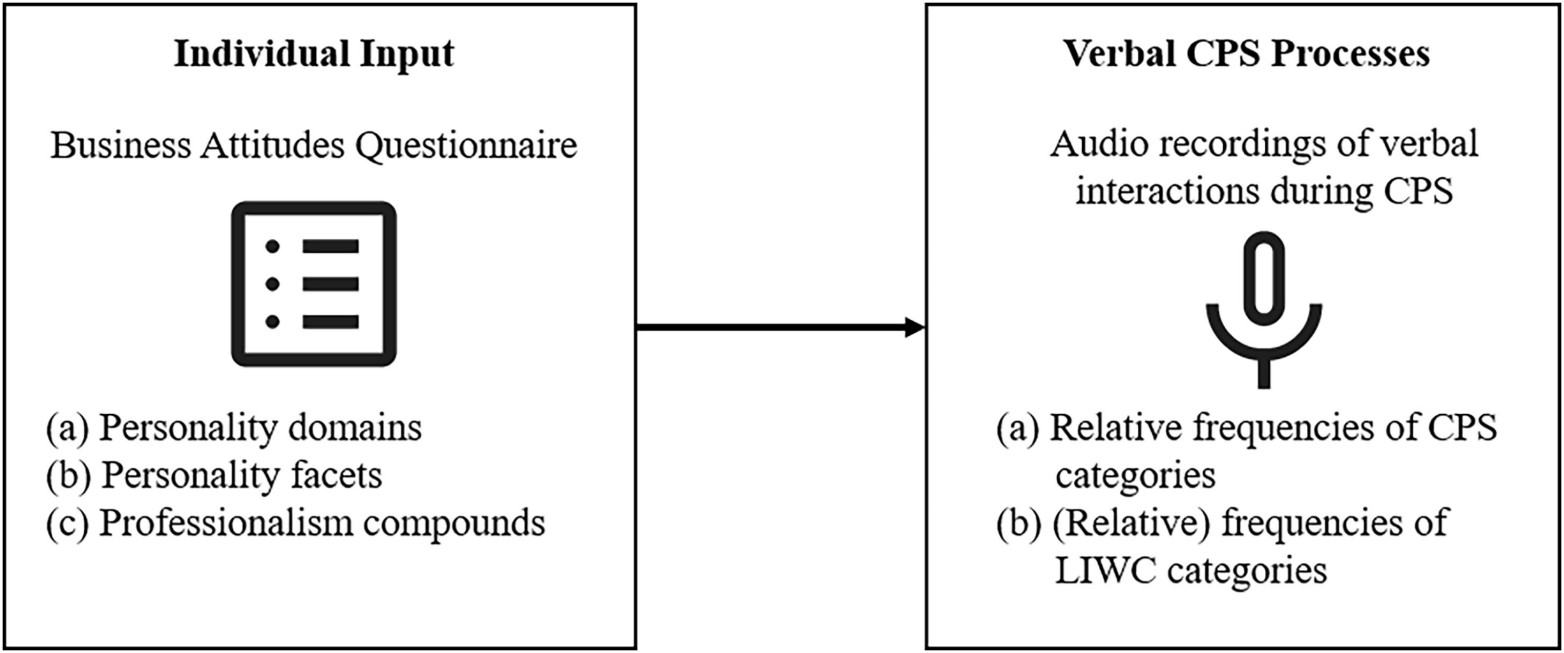
Conceptual overview of the relationship between personality traits and verbal CPS process indicators.

Figure 1 provides a conceptual overview of the study design. This study used an exploratory, deductive, quantitative content analysis design to examine associations between personality traits and verbal interaction processes during CPS. Quantitative content analysis refers to a systematic procedure in which qualitative verbal or textual material is segmented, categorized according to an explicit coding scheme, and transformed into numerical indicators for descriptive or inferential analysis (e.g., Huxley, 2020). In the present study, the qualitative source material consisted of manually transcribed team interactions during CPS. These transcripts were analyzed in two complementary ways. Utterances were coded deductively using a theory-informed CPS coding scheme. The main coding unit was the utterance, whereas the unit of analysis for statistical modeling was the participant within a problem-solving interval. For each participant and interval, the relative frequency of each CPS category was calculated and used as an outcome variable in the multilevel regression analyses. In addition, LIWC was used as a complementary dictionary-based linguistic analysis to quantify selected word-level indicators of participants’ verbal interactions.

### Context of the study

The research was conducted in the context of the STEAMS project. In this project, a CPS training program for workplace teams was created, which comprises five phases. For an in-depth overview of these phases, we refer to Buseyne et al. (2023). For the current study, we collected data during the second training phase. In this phase, learners work collaboratively on solving a variety of problems within a 30-min timeframe, without receiving prior guidance on principles of CPS. For clarity, each period devoted to solving one specific problem is hereafter referred to as a problem-solving interval. The problems are intentionally diverse, requiring a range of skills, such as verbal reasoning, spatial insight, and memory. The design promotes team interdependence through task complexity and by necessitating task division and coordination. Following this same protocol, multiple training sessions were conducted in an educational lab setting equipped for audio and video recordings.

### Sample characteristics

The study utilizes data from 36 participants, including 21 males, who were recruited through non-probability, criterion-based convenience sampling. This means that teams were not randomly selected, but were recruited based on their availability and their fit with the study’s focus on pre-existing workplace teams. Teams were eligible when they consisted of adult professionals who already worked together in an organizational context and were available to participate in the CPS training session. This sampling strategy was appropriate because the study aimed to examine verbal CPS processes in naturally occurring professional teams rather than in ad hoc or experimentally composed groups. The sample size was limited due to the use of multimodal data collection and the intensive data processing required, including detailed content analysis of interactions between team members. The participants were recruited from a mix of private and public organizations in the Flemish region and were drawn from nine pre-existing teams.

The demographic and professional characteristics of the sample are summarized in Table 2. The participants had diverse backgrounds and held various job roles. Among the participants, one individual was in the 18–24 age range, while 36% were aged 25–34, 39% were aged 35–44, and 19% were aged 45–54. One participant fell in the 55–64 age range. Regarding their work experience, participants had an average tenure of 8.5 years (SD = 6.97) in their current organization, where they participated in the study. The average duration of employment within their current team was 4.6 years (SD = 4.95). In addition, participants varied in educational background, with most holding either a master’s degree (41.67%) or a professional bachelor’s degree (33.33%). Their professional functions were also diverse, with the largest groups working in management, leadership, or coordination roles (30.56%), technical expertise roles (19.44%), and software development or engineering roles (16.67%).

**TABLE 2:** Demographic and professional characteristics of the participants.

Characteristic	Category/variable	*n*	%
Gender	Male	21	58.33
	Female	15	41.67
Age range	18–24 years	1	2.78
	25–34 years	13	36.11
	35–44 years	14	38.89
	45–54 years	7	19.44
	55–64 years	1	2.78
Education level	Master’s degree	15	41.67
	Academic bachelor’s degree	1	2.78
	Professional bachelor’s degree	12	33.33
	Associate degree	2	5.56
	Secondary education degree	6	16.67
Professional function	Management, leadership, or coordination	11	30.56
	Technical expertise	7	19.44
	Software development or engineering	6	16.67
	Human resources	4	11.11
	Content-related roles	4	11.11
	Finance	4	11.11
		**M**	**SD**
Work experience	Organizational tenure	8.50	6.97
	Team tenure	4.60	4.95

### Data collection and processing

#### Personality measures

Prior to the training, participants were asked to complete the BAQ, a personality assessment tailored to workplace contexts, which received certification from the British Psychological Society (Bogaert et al., 2014). The BAQ demonstrates strong correlations with other personality inventories, both those contextualized to the workplace and those that are not, and it has exhibited predictive capabilities for job performance (Wille et al., 2018). The BAQ gauges four facets of personality for each of the Big Five domains (see Table 1) and additionally evaluates five personality compounds (i.e., Ambitious, Critical, Results-oriented, Strategic, and Autonomous), labeled under Professionalism. The 25 BAQ facet scores and compound trait scores are computed by averaging the scores from six Likert-scale items. The Big Five domain scores are calculated by averaging the scores of the corresponding facets.

#### Verbal interaction measures

Throughout the CPS activity, team-level discussions were recorded using various individual headsets and overhead microphones. The entire process was also captured on video using the video equipment available in the educational lab. The audio recordings were transcribed manually by one of the researchers, due to the unsatisfactory performance of automatic speech recognition software in the Flemish context.

For the quantitative content analysis, the transcribed verbal interaction data were conceptualized as observable indicators of participants’ contributions to the CPS process. Following quantitative content analysis principles, the transcripts were segmented into meaningful verbal units and assigned to predefined categories representing theoretically relevant CPS behaviors (Cohen et al., 2007; Krippendorff, 2019; Neuendorf, 2017). In this study, the utterance served as the primary coding unit, because utterances capture discrete contributions to the ongoing interaction while remaining closely connected to the conversational context. After coding, these qualitative verbal units were reduced to quantifiable indicators by calculating the relative frequency of each CPS category for each participant within each problem-solving interval. This procedure made it possible to compare participants’ verbal CPS behavior across intervals and to use the coded interaction categories as outcome variables in the statistical models.

Participants’ utterances were annotated using a content analysis coding scheme for computer-supported CPS (Buseyne et al., 2024a), as visualized in Table 3. An overview of the underlying items per sub-category is provided in Supplementary Appendix 1. This coding scheme is based on previous work by Meier et al. (2007) and Sun et al. (2020). The use of the coding scheme in the context of adult CPS is discussed in more detail in Buseyne et al. (2024a). Face validity is supported by the alignment between the coding categories and the observable CPS behaviors examined. Content validity was supported by grounding the coding scheme in established CPS frameworks (Meier et al., 2007; Sun et al., 2020) and by using explicit operational categories and sub-categories.

**TABLE 3:** Coding scheme for computer-supported CPS, including categories and sub-categories.

Category	Sub-category
A: Establishing, constructing and maintaining shared knowledge and understanding	A1: Sharing knowledge and understanding of problems and solutions
	A2: Establishing common ground
B: Negotiating and coordinating for task completion and problem solving	B1: Responding to others’ ideas or proposed solutions
	B2: Monitoring execution
	B3: Time management
	B4: Technical coordination
	B5: Discussing strategies
C: Maintaining team function and organization	C1: Taking initiatives to advance collaboration processes
	C2: Coordinating task division

An independent data coder received training on the coding scheme. To ensure coding consistency, both the assigned data coder and one of the authors individually coded the transcribed data from one team. Discrepancies were discussed, leading to refinements in the coding scheme to better suit the specific context of this research. After this phase, reliability was assessed by calculating inter-rater reliability, which indicated substantial agreement at both the overall item level (κ = 0.75) and the sub-category level (κ = 0.79). The segments of the remaining teams were coded by the independent data coder using the finalized coding scheme. Finally, the relative frequency of each coding category, expressed as a percentage, was calculated for each person per problem-solving interval. The percentage for each category represents the combined total of the percentages from its underlying sub-categories.

Subsequently, the Dutch library (van Wissen and Boot, 2017) of LIWC 2015 (Pennebaker et al., 2015a,^2022^) was employed as a complementary dictionary-based linguistic analysis to evaluate the affective, social, and cognitive dimensions of participants’ interactions during each phase of the task. In our study, Utterance Count, Word Count, and Words Per Sentence, are expressed as absolute values. The remaining LIWC output variables are presented as percentages relative to the total number of words used by each individual per problem-solving interval (i.e., per problem to be solved). For instance, a value of 10.1 for personal pronouns indicates that 10.1 percent of all the words used by a person in this problem-solving interval were personal pronouns.

### Data analysis

The data analyses for this study were conducted using R (version 4.3.1; R Core Team, 2021) and started with descriptive analyses to obtain a summary of the target variables. Because the dependent variables related to verbal interaction were measured separately for multiple problem-solving intervals within each individual, the data had a hierarchical structure, with repeated observations nested within persons. Therefore, multilevel linear regression analyses were performed using the *nlme* package (version 3.1; Pinheiro and Bates, 2024), based on maximum likelihood estimation. Verbal interaction categories were regressed on (a) personality domains, (b) personality facets, and (c) personality compounds separately. Predictors were mean-centered, and the models accounted for the repeated measurements of each dependent variable per individual by treating the individual as a nested factor. Following Leyland and Groenewegen (2020), groups were not included as an additional level due to the limited number of groups. Instead, group and task variables were included as covariates in all models. Given the number of personality predictors relative to the sample size, the personality predictors were first tested individually. Subsequently, significant predictors were included in a multiple regression model. Marginal coefficients of determination for generalized mixed-effect models (Marginal *R*^2^) were calculated using the *MuMIn* package (version 1.48.4; Bartoń, 2024). This coefficient represents the proportion of variance explained by the fixed effects in the model.

## Results

Summary statistics for each of the personality domains and compounds are presented in Supplementary Table 1. Model selection for the analyses presented in this Results section is based on significant effects in single-predictor multilevel regression models, as detailed in Supplementary Appendix 2. In what follows, results are presented in three steps. First, we report the analyses for personality domains. Second, we present the facet-level analyses. Third, we report the exploratory analyses for personality compounds.

### Personality domains and verbal CPS behavior

Table 4 presents the final multilevel models for the personality domains. The table shows only the verbal interaction outcomes for which at least one domain-level predictor remained in the final model after the initial screening procedure. Although Emotional Stability was positively linked to the use of time-related words in the single-predictor regression model (see Supplementary Appendix 2), this effect did not remain significant after controlling for Openness. Extraversion was positively linked to the use of negations and Openness was significantly associated with higher engagement in sharing knowledge and understanding (A1) and the use of cognitive process words. Marginal *R*^2^ values ranged from 0.12 to 0.19, indicating moderate explanatory power. No significant effects were observed for the domains Altruism and Conscientiousness.

**TABLE 4:** Results of the multilevel regression analyses for the link between personality domains and categories of verbal interactions.

	CPS		LIWC	
Domain	A1. Sharing knowledge	Negations	Cognitive process words	Time
(Intercept)	19.91 (3.49) ***	1.91 (0.62) **	15.73 (1.27) ***	7.60 (0.88) ***
Emotional stability				0.62 (0.63)
Extraversion		0.71 (0.27) *		
**Openness**	3.79 (1.81) *		1.62 (0.66) *	**1.06 (0.52)**
Akaike information criterion (AIC)	1989.13	1143.37	1494.63	1313.31
Bayesian information criterion (BIC)	2055.58	1209.82	1561.07	1383.25
Log likelihood	-975.57	-552.69	-728.31	-636.65
σ_within_	13.18	2.33	4.79	3.29
σ_between_	0.003	0.00	0.00	0.00
*R*^2^ (marg)	0.18	0.19	0.12	0.19

Regression coefficient estimates are shown, along with the corresponding standard error in parentheses and the *p*-value.

*p < 0.05,

**p < 0.01,

***p < 0.001. Statistically significant predictor coefficients are marked in bold.

### Personality facets and verbal CPS behavior

Table 5 presents the final multilevel models for the personality facets. Although some significant single-predictor effects could be observed (see Supplementary Appendix 2), the Emotional Stability facets did not show significant associations with the verbal interaction categories after controlling for other personality facets. Similarly, no significant effect was found for the Conscientiousness facets.

**TABLE 5:** Results of the multilevel regression analyses for the link between personality facets and categories of verbal interactions.

Facet	A. Knowledge and understanding	A1. Sharing knowledge	B1. Responding	WPS	WC	Negations	Negative emotion Words	Cognitive process words	Time
(Intercept)	46.43 (4.33) ***	17.88 (3.51) ***	8.23 (1.74) ***	5.92 (0.35) ***	207.38 (24.34) ***	1.81 (0.66) **	0.27 (0.21)	15.72 (1.25) ***	7.53 (0.89) ***
Relaxed								-0.02 (0.84)	
Optimistic									0.65 (0.39)
Stress Resistant								1.13 (0.91)	
Decisive						0.16 (0.42)			
Leading						-0.16 (0.34)			
Persuasive						0.96 (0.50)			
Motivating				-0.45 (0.18) *		0.01 (0.42)		-1.27 (0.60) *	
Abstract								0.86 (0.39) *	
Innovative									0.04 (0.57)
Change- oriented	4.77 (1.82) *	3.36 (1.51) *	-1.65 (0.73) *						0.73 (0.49)
Open- minded									0.56 (0.55)
People- oriented					27.14 (12.79) *				
**Helpful**		**-3.86 (2.08)**					0.27 (0.12) *		
AIC	2088.87	1984.26	1644.36	799.56	2859.92	1145.61	620.88	1488.97	1312.23
BIC	2155.32	2054.21	1710.80	866.01	2926.36	1222.54	687.33	1565.91	1389.17
Log likelihood	-1025.44	-972.13	-803.18	-380.78	-1410.96	-550.80	-291.44	-722.49	-634.12
σ_within_	16.18	13.00	12.68	1.10	75.13	2.31	0.80	4.67	3.25
σ_between_	0.001	0.001	0.00	0.39	26.32	0.00	0.00	0.00	0.00
*R*^2^ (marg)	0.18	0.20	0.30	0.25	0.30	0.20	0.11	0.18	0.20

Regression coefficient estimates are shown, along with the corresponding standard error in parentheses and the *p*-value.

*p < 0.05,

**p < 0.01,

***p < 0.001. Statistically significant predictor coefficients are marked in bold.

The Motivating facet of Extraversion was negatively associated with words per sentence and cognitive process words. In addition, it is worth noting that the effect of Persuasive on the use of negation words approached significance.

For Openness, the Abstract facet was associated with increased use of cognitive process words, and the Change-Oriented facet of Openness was positively associated with overall knowledge construction behaviors (A) and sharing knowledge. However, Change-Oriented individuals were less likely to engage in responding to others’ ideas (B1).

The People-Oriented facet of Altruism was linked to higher overall word count. Last, the Helpful facet of Altruism was positively related to the use of negative emotion words.

Marginal *R*^2^ values for these models ranged from 0.11 to 0.30, reflecting moderate to good explanatory power.

### Personality compounds and verbal CPS behavior

Table 6 presents the final multilevel models for the BAQ personality compounds. The results of personality compounds suggested several additional associations. Ambitious was positively linked to constructing and maintaining shared knowledge (A) and negatively to negotiation and coordination (B). Critical was negatively associated with time management behaviors (B3). The Results-Oriented compound was positively linked to higher word count, while Strategic individuals used more cognitive process words. Marginal *R*^2^ values ranged from 0.15 to 0.33, reflecting moderate explanatory power across models.

**TABLE 6:** Results of the multilevel regression analyses for the link between personality compounds and categories of verbal interactions.

Compound	A. Knowledge and understanding	B. Negotiating and coordinating	B3. Time management	Word count	Cognitive process words
(Intercept)	45.18 (4.44) ***	45.74 (4.41) ***	4.50 (1.19) ***	186.37 (24.06) ***	15.19 (1.32) ***
Ambitious	6.27 (2.40) *	-6.41 (2.39) *			
Critical			-1.58 (0.68) *		0.69 (0.77)
Results-oriented				41.92 (16.19) *	
**Strategic**				**12.01 (15.16)**	1.78 (0.76) *
AIC	2088.88	2085.93	1427.80	2854.57	1491.83
BIC	2155.33	2152.37	1494.25	2924.52	1561.77
Log likelihood	-1025.44	-1023.96	-694.90	-1407.29	-725.92
σ_within_	16.18	16.08	4.08	74.96	4.74
σ_between_	0.001	0.00	0.94	21.15	0.00
*R*^2^ (marg)	0.18	0.23	0.15	0.33	0.15

Regression coefficient estimates are shown, along with the corresponding standard error in parentheses and the *p*-value.

*p < 0.05,

**p < 0.01,

***p < 0.001. Statistically significant predictor coefficients are marked in bold.

While all personality predictors included in the models visualized above were individually significant in single-predictor analyses, several personality predictors did not retain their significance in the multiple regression models. For instance, Openness and its facets remained significant in relation to knowledge-sharing behaviors, but other personality facets such as Abstract and Motivating, although significant in isolation, did not exhibit significant effects once other predictors were included in the model.

## Discussion

The study was exploratory, involving a limited number of independent participants, and examining multiple personality constructs and verbal interaction indicators. Accordingly, the observed associations should not be interpreted as evidence of stable psychological mechanisms or as definitive support for trait-based explanations of CPS behavior. Rather, the interpretations offered below are possible explanations that may help guide future research which could experimentally test a specific relationship between personality and CPS behavior.

### Personality domains and facets in relation to verbal CPS behavior

The first two research questions in this study focused on examining how the Big Five personality domains and their underlying facets relate to verbal CPS behavior. No significant relationships were found between Emotional Stability and any verbal CPS behavior categories. Similarly, no significant relationships emerged for Conscientiousness, either at the domain or facet level. A possible explanation for this finding lies in the short-term nature of the collaboration examined in this study, as previous research by Jolić Marjanović et al. (2024) suggests that Conscientiousness may play a more critical role in long-term collaborations.

Extraversion was not significantly related to knowledge sharing or coordinating behaviors. In addition, there was no significant relationship found with word count. However, Extraversion was positively associated with the use of negation words. On the facet level of Extraversion too, various significant relationships could be identified with the use of negations in single personality predictor models. However, in the combined models, these facets were no longer significantly associated with negations. One possible explanation lies in the larger effect of Persuasive, a facet of Extraversion. Persuasive individuals often thrive in dynamic interactions and feel at ease during negotiations, which may explain their higher use of negations and expressions of opposition.

One of the facets of Extraversion, Motivating, which reflects a tendency to inspire others, foster enthusiasm, and energize task engagement, was found to be negatively associated with both words per sentence and cognitive process words. This indicates that individuals high in Motivating may adopt a more concise communication style, favoring brevity over detailed or analytical expressions. Additionally, while this style may suggest a reduced contribution to the reflective or analytical dimensions of the CPS process, this was not evident in the results for CPS categories of verbal interaction. The results at the facet level suggest the potential value of examining personality facets independently, as they may reveal more nuanced and, at times, contrasting patterns of behavior compared to overarching domains.

Openness was positively associated with higher engagement in knowledge-sharing behaviors and the use of cognitive process words. This finding is consistent with the domain’s well-established role in fostering creativity, intellectual exploration (DeYoung et al., 2010), and its contribution to intellectual and creative achievements (Jolić Marjanović et al., 2024). Consistent with Lin et al. (2015), our results are compatible with a possible positive association between Openness and knowledge-sharing behaviors in team contexts, suggesting that Openness may be relevant for generating and integrating ideas during CPS. Additionally, these findings are broadly in line with those of Curşeu et al. (2019), who linked Openness to creative problem-solving and reflective communication, but replication in larger samples is needed before drawing stronger theoretical conclusions.

Facet-level analyses indicated that the associations involving Openness were largely attributable to its Change-Oriented facet, which reflects a preference for change, novelty, and variety over routine. In this study, individuals with a change-oriented personality demonstrated a positive association with behaviors related to establishing, constructing, and maintaining shared knowledge and understanding in CPS. However, they were negatively associated with interactions involving responding to others’ ideas or proposed solutions. This pattern may suggest that while change-oriented individuals excel at initiating and fostering shared understanding, they may be less inclined to engage in behaviors that involve directly addressing or elaborating on the contributions of others.

At the domain level, Altruism was not significantly associated with any verbal interaction categories. However, individuals scoring higher on the People-Oriented facet of Altruism exhibited a significantly higher overall word count, suggesting greater overall engagement in interactions. A possible interpretation is that participants scoring higher on People-Oriented were more verbally engaged in the group interaction, which would be compatible with the social orientation captured by this facet. Additionally, individuals scoring higher on the Helpful facet of Altruism used significantly more negative emotion words. However, it is important to note that LIWC does not indicate the pragmatic function of these words in context.

### Personality compounds in relation to verbal CPS behavior

The third research question sought to explore the relationship between the personality compounds Ambitious, Critical, Results-Oriented, Strategic, and Autonomous and their relation with verbal behaviors during CPS. The results for the BAQ personality compounds provide tentative and preliminary indications of additional associations with CPS interactions beyond the Domains and Facets. Four compounds, i.e., Ambitious, Critical, Result-Oriented, and Strategic, were significantly associated with at least one of the categories. Given the exploratory nature of these compound-level analyses, these associations should not be taken as conclusive evidence of stable personality-based mechanisms.

Participants scoring higher on Ambitious showed a tentative positive association with establishing, constructing, and maintaining shared knowledge and understanding. However, their reduced engagement in negotiating and coordinating for task completion and problem solving suggests a focus on goal-setting and solution generation rather than the integration of diverse perspectives. This observation aligns with the findings for Change-Oriented facet of Openness.

Participants scoring higher on Critical showed a tentative negative association with utterances related to time management. One possible interpretation is that participants scoring higher on Critical may have focused less on monitoring timelines during the task. Instead, Critical individuals may prioritize evaluating the quality and validity of ideas over monitoring timelines.

Results-Oriented individuals, who strive for success, seek recognition, and are driven by competition, demonstrated a higher overall word count. This may indicate greater participation and interaction during the CPS activity (Tausczik and Pennebaker, 2010), which would be compatible with the achievement-oriented nature of the construct.

Lastly, strategic showed a tentative positive association with cognitive process words. One possible interpretation is that participants scoring higher on Strategic used more language related to reasoning, possibility, or reflection during the CPS task. This would be conceptually compatible with the forward-looking and analytical nature of the compound.

### Relative contributions of domains, facets, and compounds

For the analyses conducted at the personality domain level, where task and team were controlled for, the marginal *R*^2^ values ranged from 0.12 to 0.19 after accounting for task and team effects. Marginal *R*^2^ values for facet models ranged from 0.11 to 0.30, indicating moderate to good explanatory power. The variability suggests that the models may offer a mixed level of detail and precision in predicting specific behaviors. These nuanced facet-level findings align with recommendations of Burch and Anderson (2008) and previous research by Nielsen and Kajonius (2024) who argued that facets offer more precise predictors of life outcomes than broader traits.

The models including personality compounds showed similar marginal *R*^2^ values, ranging from 0.15 to 0.33. This may suggest that personality compounds, due to their alignment with workplace behaviors, may offer a more context-sensitive account of some verbal CPS interactions compared to the more generalized domains. This point to the potential value of considering contextually relevant personality constructs, like compounds, when examining personality differences in complex team interactions such as in CPS. The results align with Wille et al. (2018), who highlighted the predictive power of the BAQ’s professionalism-related compounds for job performance. Furthermore, this is broadly in line with the assertion by Stanek and Ones (2018) that, “if leveraged appropriately, thorough and up-to-date construct taxonomies and measure compendia can better inform organizational practices, as well as academic research and theory-building” (p. 3).

### Strengths and limitations

This study offers several strengths that contribute initial insights into the relationship between personality and CPS processes. By examining personality traits across domains, facets, and compounds, this study provides a more nuanced understanding of individual differences and their association with verbal CPS behavior. Furthermore, rather than relying on self-reports or peer-reports of behavioral indicators, this study utilizes a more rigorous and innovative approach by combining objective methods and diverse data sources, using content analysis and LIWC to analyze transcripts of team members’ verbal interactions. Another strength of this study is its focus on pre-existing adult teams from diverse professional contexts. In contrast, previous research on CPS processes and outcomes has predominantly relied on student samples, limiting the generalizability of findings to real-world collaborative environments.

Besides these strengths, the study has some limitations which need to be considered. For instance, several aspects limit the generalizability of our findings. First, the study’s sample size was limited in relation to the broad analytical scope. The repeated-observation structure of the data does not fully offset this limitation. Although each participant contributed multiple observations across problem-solving intervals, the personality predictors were measured at the participant level. This is particularly important because the study explored a broad set of personality domains, facets, compounds, and verbal interaction indicators. As a result, the analyses are vulnerable not only to limited statistical power, but also to model instability, overfitting, multiplicity, and capitalization on chance. Therefore, it is crucial to interpret the findings as tentative patterns that require replication analysis in future research.

Second, although the CPS task design allowed to include participants from diverse backgrounds, the CPS task was not representative of participants’ daily work activities. Thus, the results may not be directly applicable to other settings or populations and the findings should be interpreted as preliminary.

A third limitation concerns the exploratory model-selection procedure. Because the number of personality predictors was large relative to the number of independent participants, we did not estimate models including all personality facets or compounds simultaneously. Instead, we first examined predictors in individual models and then included statistically significant predictors in multivariable follow-up models. Although this procedure was chosen to avoid overparameterized models, it also introduces limitations. In particular, this can increase the risk of Type I error inflation (e.g., Ranganathan et al., 2016).

An additional limitation concerns the team-based structure of the data. Although the analyses accounted for repeated observations within individuals and included task and team indicators as covariates, the limited number of teams did not allow us to estimate a full multilevel model with team-level random effects. This relatively small number of teams reflects the fact that the study was conducted within an authentic training trajectory, in which opportunities to include additional teams were constrained. This limitation is important because verbal CPS behavior is produced within collaborative interactions. Consequently, the observed associations between individual personality scores and verbal behavior should not be interpreted as purely individual-level personality effects. Some associations may partly reflect unmodeled team-level influences. For example, a participant’s word count, knowledge-sharing behavior, or use of cognitive process words may depend not only on their personality, but also on whether the specific team context encouraged more or less participation. Future research is needed to replicate these findings in larger and more varied CPS contexts. In particular, future research should include a larger number of teams to disentangle individual-level personality associations from team-level influences and team composition effects.

### Directions for future research

Future research could build on the present findings by examining a selection of theoretically selected personality–behavior associations. For example, the tentative associations observed for Openness, the Change-Oriented facet, Ambitious, Results-Oriented, and Strategic could be examined in larger samples to determine whether these patterns are stable across different CPS tasks, team compositions, and professional contexts. Such studies should also include a sufficient number of teams to distinguish individual-level personality associations from team-level influences, such as team norms, role distribution, leadership, or prior collaboration history.

This study examined the associations between personality traits and CPS interactions linearly. However, the potential curvilinearity of these relationships was not assessed. Such analyses could provide a more nuanced understanding of how personality traits are associated with CPS behaviors. Specifically, the association between certain traits and verbal interaction might not be straightforwardly linear but could instead follow a more complex pattern, where both very high and very low levels of a trait might lead to different interaction dynamics (e.g., Le et al., 2011). Future research could consider these non-linear relationships to better capture how personality may relate to CPS. Additionally, our study focused on the individual effects of personality traits on CPS interactions without accounting for possible interaction effects among the personality dimensions. Future research could investigate these potential interactions among different traits to gain a more comprehensive understanding of how personality may be associated with CPS behaviors.

Future research could also move beyond the frequency of verbal behaviors and examine their timing, sequence, and interactional function. For example, the present study suggests that some personality constructs may be associated with knowledge sharing, cognitive process words, or word count. However, these indicators do not reveal whether contributions were taken up by other team members, whether they advanced the team’s problem-solving process, or how they unfolded within the interaction. Sequential or process-oriented approaches could therefore help clarify whether personality is associated not only with how much team members contribute, but also with when, how, and in response to whom they contribute.

Furthermore, our study employed univariate regression models to examine each category of verbal interaction separately. Future research could benefit from using alternative analysis methods, such as epistemic network analysis (Swiecki et al., 2020). Epistemic network analysis allows researchers to not only focus on the frequency of individual parameters but also to analyze the co-occurrence of these parameters within specific time frames.

In the longer term, and only after more robust evidence has accumulated, this line of research may inform the development of CPS or workplace tools that integrate multiple forms of assessment, including verbal interaction data. For example, future studies could examine whether combining self-report measures with interaction-based indicators provides useful feedback for teams. The current study should therefore be viewed as a preliminary step toward identifying personality–interaction patterns that may eventually inform research on team feedback, training, or collaborative support tools.

Similarly, the methodological approach used in this study may also inform future research on collaborative tools and platforms that integrate multiple forms of assessment. For example, many existing tools for team feedback rely primarily on self-reported data (e.g., Phielix et al., 2011), whereas future research could examine whether verbal interaction indicators provide additional information about team processes. Related work on automated or semi-automated analysis of collaborative problem solving suggests that interaction data may eventually be useful for supporting feedback systems (Stewart et al., 2023). Further work is needed to determine whether, how, and under which conditions verbal interaction indicators can provide valid, useful, and actionable feedback for teams.

## Data Availability

The raw data supporting the conclusions of this article will be made available upon reasonable request and subject to appropriate data sharing agreements.
