# Supplementary material for: Personality’s voice: how personality traits shape verbal processes in collaborative problem-solving
Source: Front Psychol. 2026 Jul 22;17:1830668. doi: 10.3389/fpsyg.2026.1830668 (PMC13437450; doi:10.3389/fpsyg.2026.1830668)
Supplement: Supplementary file 1 [file Table_1.docx]

**Appendices**

**Appendix A**

Appendix A includes a detailed overview of the (a) CPS sub-categories and items (Table A1) and (b) the Business Attitudes Questionnaire (BAQ) personality framework used in this study (Table A2).

**Table A1**

*Detailed Overview of Sub-Categories and Their Corresponding Items*

| Sub-category | Item |
| --- | --- |
| A1: Sharing knowledge and understanding of problems and solutions | Proposing appropriate solutions or introducing new appropriate information related to the problem  Talking about givens and constraints of a specific task  Building on others' ideas to improve solutions |
| A2: Establishing common ground | Asking for further clarification  Giving feedback on the understanding of what the other is saying and asking questions  Eliciting feedback from the one who is listening  Clarifying any information needs and responding to questions  Evoking turn-taking by means of explicit handovers  Repairing misunderstandings |
| B1: Responding to others' ideas or proposed solutions | Providing reasons to support a potential solution  Questioning, correcting or pointing out others' mistakes  Confirming to support a potential solution |
| B2: Monitoring execution | Talking about or discussing the results |
| B3: Time management | Monitoring time |
| B4: Technical coordination | Using the technical tools |
| B5: Discussing strategies | Discussing the general group strategies |
| C1: Taking initiatives to advance collaboration processes | Asking if others have suggestions  Asking to take action before anyone on the team asks for help  Complimenting or encouraging others  Apologizing for one’s mistake(s).  Proposing to ask or asking for help outside of the group |
| C2: Coordinating task division | Defining (sub)tasks and talking about the adoption of these tasks |

**Table A2**

*Descriptions of the BAQ Personality Domains, Facets, and Compounds*

| Facet | Low Level Description | High Level Description |
| --- | --- | --- |
| **Emotional Stability** | | |
| Relaxed | Anxious or guilty in the event of failure, worried, lacking in calmness, nervous | Free from anxiety, maintains a calm attitude in the event of failure, calm and relaxed |
| Optimistic | Expects things to go wrong, worries about how things will turn out, pessimistic | Confident about the outcome of things, does not worry, remains cheerful |
| Stress-resistant | Sensitive to stress, copes poorly with tension and pressure, quickly affected by situations | Not very sensitive to stress, not particularly bothered by tension and pressure, not easily affected by situations |
| Decisive | Hesitates over decisions, needs time to reach conclusions | Makes decisions quickly, based on (in)sufficient data, draws firm conclusions |
| **Extraversion** | | |
| Leading | Lets others take the lead, gives away the initiative, doesn’t like giving instructions | Likes to lead, takes the initiative, gives others instructions |
| Communicative | Not fond of speaking, has trouble to keep the conversation going, is inarticulate | Is fond of speaking, keeps conversation going, is articulate |
| Persuasive | Poor salesperson, uncomfortable in negotiations, not convincing | Able to sell, comfortable in negotiations, convincing |
| Motivating | Uninspiring, lacks a motivating influence, leaves others to fend for themselves | Inspires others, has a motivating influence, inspires enthusiasm for the task |
| **Openness** | | |
| Abstract | Concrete, both feet on the ground, practical-minded | Theoretical, intellectually curious, likes complex, abstract things |
| Innovative | Lacks inventiveness and creativity, rarely thinks of new ways of seeing things | Is creative, generates new ideas and thinks of original ways of seeing things |
| Change-oriented | Prefers routine, needs security, prefers regularity to variety | Likes change, tries out new things, prefers variety to regularity |
| Open-minded | Does not see many possibilities, has trouble thinking up alternatives and options | Sees various possibilities, thinks up alternatives and options |
| **Altruism** | | |
| People-oriented | Enjoys being alone, is not very fond of company, is focused on him/herself, doesn’t need company | Enjoys group situations, is fond of company, is focused on others, seeks out company |
| Cooperating | Rarely consults or involves others, does not seek cooperation, places own interests above those of the group | Consults and involves others, seeks cooperation, places group’s interests above his/her own |
| Helpful | Self-involved, lacks a helpful attitude, isn’t concerned about others, lacks considerateness | Helps when there are problems, gives advice, is considerate |
| Socially Confident | Finds it hard to establish contact, doesn’t always get along with people, unfriendly, unpleasant | Establishes contact easily, cheerful, gets along with people, friendly, pleasant, spontaneous |
| **Conscientiousness** | | |
| Organized | Does not work to a plan, sets insufficient deadlines, overlooks routine tasks | Plans carefully in the light of priorities, sets deadlines, keeps routine tasks in mind |
| Meticulous | Not very methodical or meticulous, has little eye for detail | Works methodically and meticulously, pays attention to details |
| Rational | Pays little attention to facts, relies on intuition, tends not to quantify, speaks or acts first and thinks afterwards | Sticks to the facts, evaluates and measures, quantifies, thinks twice before speaking or acting |
| Persevering | Loses heart quickly, gives up when facing opposition, rarely sees tasks through to a successful conclusion | Perseveres despite setback, keeps trying, persists in the face of opposition |
| Professionalism Compounds | | |
| Ambitious | Not very career-minded, unambitious, sets moderate objectives | Career-minded, ambitious, sets difficult objectives, aims high |
| Critical | Not very critical in his/her approach, accepts information or ideas from others without question | Examines information critically, identifies potential drawbacks and limitations |
| Results-oriented | Not very results-oriented, feels little need to achieve results, lacks competitiveness | Likes to achieve results, wants to stand out, is competitive |
| Strategic | Sets short-term objectives, looks at things from an operational or short-term perspective | Sets long-term objectives, looks at things from a strategic or long-term perspective |
| Autonomous | Adapts to the situation, adapts to the circumstances, does not show own approach or opinion | Influences the situation, marks the situation with his/her personality, has his/her own approach and opinion |

**Appendix B**

Appendix B presents an overview of the statistical analyses conducted in this study to explore the relationship between personality traits and verbal CPS behaviors. The appendix includes descriptive statistics for personality domains, facets, and compounds (Table B1), as well as results from single and multilevel regression models linking these personality traits to specific CPS categories and linguistic markers (Tables B2 through B7). The analyses provide detailed insights into how personality dimensions influence various aspects of CPS, such as knowledge sharing, negotiation, and coordination, alongside linguistic indicators like word count, negations, and cognitive process word usage.

**Table B1**

*Summary Statistics of the Personality Domains, Facets, and Compounds*

|  | ***M*** | ***SD*** | **Median** | **Minimum** | **Maximum** |
| --- | --- | --- | --- | --- | --- |
| **Emotional Stability** | 3.62 | 0.49 | 3.67 | 2.33 | 4.50 |
| Relaxed | 3.29 | 0.72 | 3.33 | 1.50 | 4.67 |
| Optimistic | 3.90 | 0.74 | 3.83 | 2.17 | 5.00 |
| Stress-resistant | 3.52 | 0.64 | 3.58 | 2.17 | 4.83 |
| Decisive | 3.76 | 0.58 | 3.83 | 2.50 | 5.00 |
| **Extraversion** | 3.40 | 0.59 | 3.38 | 1.92 | 4.79 |
| Leading | 3.27 | 0.76 | 3.50 | 1.50 | 4.67 |
| Communicative | 3.37 | 0.73 | 3.25 | 2.17 | 4.83 |
| Persuasive | 3.47 | 0.67 | 3.50 | 1.67 | 5.00 |
| Motivating | 3.51 | 0.65 | 3.50 | 2.00 | 5.00 |
| **Openness** | 3.48 | 0.56 | 3.42 | 2.21 | 4.75 |
| Abstract | 2.93 | 0.97 | 3.17 | 1.00 | 5.00 |
| Innovative | 3.43 | 0.67 | 3.33 | 2.17 | 5.00 |
| Change-oriented | 3.85 | 0.61 | 4.00 | 2.17 | 5.00 |
| Open minded | 3.73 | 0.54 | 3.83 | 2.67 | 4.83 |
| **Altruism** | 3.82 | 0.48 | 3.92 | 2.54 | 4.63 |
| People-oriented | 3.37 | 0.69 | 3.42 | 2.00 | 5.00 |
| Cooperating | 3.79 | 0.61 | 3.83 | 1.50 | 5.00 |
| Helpful | 4.27 | 0.51 | 4.33 | 2.33 | 5.00 |
| Socially Confident | 3.85 | 0.70 | 4.00 | 1.67 | 5.00 |
| **Conscientiousness** | 3.72 | 0.49 | 3.75 | 2.46 | 4.58 |
| Organised | 3.48 | 0.76 | 3.33 | 1.67 | 5.00 |
| Meticulous | 3.55 | 0.78 | 3.50 | 1.83 | 5.00 |
| Rational | 3.78 | 0.64 | 4.00 | 2.33 | 5.00 |
| Persevering | 4.05 | 0.46 | 4.00 | 2.50 | 4.83 |
| Compounds |  |  |  |  |  |
| Ambitious | 3.46 | 0.66 | 3.50 | 2.17 | 5.00 |
| Critical | 4.07 | 0.62 | 4.00 | 2.33 | 5.00 |
| Result oriented | 3.63 | 0.55 | 3.67 | 2.50 | 4.83 |
| Strategic | 3.63 | 0.66 | 3.67 | 1.17 | 5.00 |
| Autonomous | 3.89 | 0.48 | 3.83 | 3.00 | 5.00 |

**Table B2**

*Overview of Predictors in Single Regression Models for the Link Between Personality and CPS Categories of Verbal Interactions*

| Predictor | CPS Category | | | | | | | | | | | |
| --- | --- | --- | --- | --- | --- | --- | --- | --- | --- | --- | --- | --- |
|  | A. Knowledge & Understanding | A1. Sharing Knowledge | A2. Establishing Common Ground | B. Negotiating & Coordinating | B1. Responding | B2. Monitoring Execution | B3. Time Management | B4. Technical Coordination | B5. Discussing Strategies | C. Team Functioning | C1. Advancing Collaboration | C2. Task Division |
| **Emotional Stability** | 2.83 (2.73) | 1.75 (2.30) | 1.10 (2.11) | -3.13 (2.71) | -1.21 (1.09) | -1.86 (1.52) | -0.66 (0.85) | 1.17 (1.70) | -0.56 (1.77) | -0.35 (1.61) | 0.15 (0.49) | -0.53 (1.56) |
| Relaxed | 2.36 (1.73) | 2.85 (1.40) | -0.49 (1.34) | -1.76 (1.72) | -1.37 (0.69) | -1.54 (0.97) | 0.22 (0.54) | 0.1 (1.08) | 0.83 (1.12) | -0.28 (1.03) | -0.17 (0.31) | -0.13 (0.99) |
| Optimistic | -1.46 (1.8) | -1.59 (1.51) | 0.13 (1.39) | 1.21 (1.79) | 0.18 (0.72) | -0.34 (1.01) | -0.5 (0.56) | 1.52 (1.12) | 0.38 (1.17) | 0.32 (1.07) | 0.06 (0.33) | 0.11 (1.03) |
| Stress-resistant | 2.66 (1.87) | 2.27 (1.55) | 0.40 (1.45) | -2.65 (1.86) | -1.03 (0.75) | -1.04 (1.05) | -0.54 (0.58) | 0.32 (1.17) | -0.37 (1.22) | -0.50 (1.11) | -0.07 (0.34) | -0.37 (1.07) |
| Decisive | 1.74 (2.01) | -0.68 (1.70) | 2.41 (1.54) | -2.89 (1.99) | 0.17 (0.80) | -0.36 (1.12) | -0.48 (0.62) | 0.16 (1.25) | -2.37 (1.29) | -0.20 (1.19) | 0.55 (0.35) | -0.69 (1.14) |
| **Extraversion** | -1.13 (1.91) | -1.91 (0.24) | 0.78 (1.47) | 0.83 (1.90) | 0.91 (0.76) | 0.55 (1.07) | -0.38 (0.59) | 1.27 (1.19) | -1.50 (1.23) | -0.53 (1.13) | 0.25 (0.34) | -0.784 (1.09) |
| Leading | -0.48 (1.39) | 2.85 (1.40) | 0.45 (1.06) | 0.13 (1.37) | 0.87 (0.55) | 0.30 (0.77) | -0.38 (0.43) | 0.71 (0.86) | -1.37 (0.89) | -0.34 (0.81) | 0.3 (0.25) | -0.65 (0.78) |
| Communicative | -0.08 (1.60) | -1.59 (1.51) | 0.88 (1.23) | 0.31 (1.59) | 0.36 (0.64) | 0.41 (0.89) | -0.35 (0.49) | 0.34 (0.99) | -0.44 (1.03) | -0.37 (0.94) | -0.04 (0.29) | -0.32 (0.91) |
| Persuasive | -1.17 (1.99) | 2.27 (1.55) | 0.47 (1.53) | 1.15 (1.97) | 0.54 (0.79) | 0.54 (1.11) | -0.29 (0.62) | 1.21 (1.24) | -0.83 (1.28) | -0.73 (1.17) | 0.07 (0.36) | -0.8 (1.13) |
| Motivating | -2.48 (1.93) | -0.68 (1.70) | 0.58 (1.49) | 1.61 (1.92) | 0.97 (0.77) | 0.54 (1.08) | -0.01 (0.61) | 2.16 (1.20) | -2.05 (1.24) | -0.28 (1.14) | 0.41 (0.35) | -0.71 (1.10) |
| **Openness** | 4.41 (2.23) | 3.79 (1.81) * | 0.62 (1.74) | -1.65 (2.24) | -1.80 (0.89) | -2.12 (1.25) | -0.69 (0.70) | 1.07 (1.40) | 1.88  (1.45) | -1.13 (1.33) | -0.45 (0.40) | -0.594 (1.28) |
| Abstract | 1.88 (1.27) | 2.85 (1.40) | 0.37 (0.98) | -0.47 (1.27) | -0.98 (0.51) | -0.72 (0.71) | -0.02 (0.40) | 0.03 (0.80) | 1.19 (0.82) | -0.59 (0.75) | -0.25 (0.23) | -0.27 (0.73) |

**Table B2 (Continued)**

|  | A. Knowledge & Understanding | A1. Sharing Knowledge | A2. Establishing Common Ground | B. Negotiating & Coordinating | B1. Responding | B2. Monitoring Execution | B3. Time Management | B4. Technical Coordination | B5. Discussing Strategies | C. Team Functioning | C1. Advancing Collaboration | C2. Task Division |
| --- | --- | --- | --- | --- | --- | --- | --- | --- | --- | --- | --- | --- |
| Innovative | 3.81 (1.94) | -1.59 (1.51) | 0.88 (1.51) | -0.99 (1.94) | -0.97 (0.78) | -1.45 (1.09) | -0.54 (0.61) | 1.17 (1.22) | 0.82 (1.26) | -1.49 (1.15) | -0.28 (0.35) | -1.16 (1.11) |
| Change-oriented | 4.77 (1.82) * | 4.03 (1.48) * | 0.75 (1.43) | -3.53 (1.83) | -1.65 (0.73) * | -2.02 (1.03) | -0.46 (0.58) | -0.1 (1.16) | 0.7 (1.2) | -0.17 (1.09) | -0.12 (0.34) | -0.04 (1.05) |
| Open-minded | -0.36 (2.32) | -0.68 (1.7) | -0.99 (1.78) | 1.48 (2.29) | -0.45 (0.92) | -1.39 (1.28) | -1.36 (0.68) | 2.9 (1.43) | 1.77 (1.48) | -0.49 (1.36) | -0.47 (0.41) | 0.07 (1.31) |
| **Altruism** | -1.24  (2.54) | -2.99 (0.16) | 1.75 (1.96) | 0.44 (2.53) | 0.96 (1.02) | 1.47 (1.41) | -0.21 (0.79) | -0.02 (1.58) | -1.72 (1.64) | 0.05 (1.50) | 0.31 (0.45) | -0.40 (1.45) |
| People-oriented | 0.71 (2.1) | 2.85 (1.40) | 1.17 (1.61) | 0.06 (2.08) | -0.12 (0.84) | 0.7 (1.17) | 0.01 (0.65) | -0.32 (1.31) | -0.21 (1.35) | -0.3 (1.24) | 0.01 (0.38) | -0.45 (1.19) |
| Cooperating | -0.63 (2.12) | -1.59 (1.51) | 0.85 (1.63) | 0.16 (2.1) | 0.5 (0.85) | 1.2 (1.18) | -0.19 (0.66) | 0.5 (1.32) | -1.85 (1.36) | 0.08 (1.25) | 0.32 (0.38) | -0.33 (1.2) |
| Helpful | -3.61 (2.54) | -4.96 (2.04) * | 1.35 (1.97) | 1.37 (2.54) | 1.91 (1.01) | 1.37 (1.42) | -0.33 (0.8) | 0.03 (1.59) | -1.51 (1.65) | 1.08 (1.5) | 0.53 (0.45) | 0.46 (1.45) |
| Socially Confident | -0.71 (1.73) | -0.68 (1.70) | 1.25 (1.33) | 0.04 (1.71) | 0.66 (0.69) | 0.81 (0.96) | -0.12 (0.54) | -0.17 (1.07) | -1.11 (1.11) | -0.24 (1.02) | 0.11 (0.31) | -0.43 (0.98) |
| **Conscien-tiousness** | -0.15  (2.57) | -1.23 (2.15) | 1.07 (1.97) | -0.52 (2.54) | 0.63 (1.02) | -1.35 (1.42) | -0.91 (0.78) | 0.86 (1.59) | 0.23 (1.65) | 1.46 (1.50) | -0.47 (0.45) | 1.85 (1.45) |
| Organised | 0.06 (1.64) | 2.85 (1.40) | 0.76 (1.25) | -0.74 (1.62) | 0.6 (0.65) | -0.79 (0.91) | -0.2 (0.51) | 0.09 (1.01) | -0.44 (1.05) | 0.66 (0.96) | -0.27 (0.29) | 0.88 (0.92) |
| Meticulous | -1.01 (1.67) | -1.59 (1.51) | 0.60 (1.29) | 0.71 (1.66) | 0.22 (0.67) | -0.28 (0.93) | -0.26 (0.52) | 0.82 (1.04) | 0.18 (1.08) | 1.04 (0.98) | -0.23 (0.3) | 1.18 (0.95) |
| Rational | 1.83 (2.18) | 2.27 (1.55) | 0.13 (1.69) | -0.89 (2.17) | -0.46 (0.87) | -1.79 (1.21) | -1.15 (0.65) | 0.43 (1.36) | 2.07 (1.4) | 0.37 (1.29) | -0.66 (0.38) | 1.06 (1.24) |
| Persevering | -1.18 (2.95) | -0.68 (1.7) | 1.08 (2.27) | -0.92 (2.93) | 1.57 (1.17) | -0.51 (1.64) | -1.31 (0.9) | 0.97 (1.84) | -1.66 (1.9) | 1.73 (1.74) | 0.34 (0.53) | 1.38 (1.67) |

**Table B2 (Continued)**

|  | A. Knowledge & Understanding | A1. Sharing Knowledge | A2. Establishing Common Ground | B. Negotiating & Coordinating | B1. Responding | B2. Monitoring Execution | B3. Time Management | B4. Technical Coordination | B5. Discussing Strategies | C. Team Functioning | C1. Advancing Collaboration | C2. Task Division |
| --- | --- | --- | --- | --- | --- | --- | --- | --- | --- | --- | --- | --- |
| Compounds |  |  |  |  |  |  |  |  |  |  |  |  |
| Ambitious | 6.27 (2.40) * | 3.43 (1.99) | 2.85 (1.87) | -6.41 (2.39) * | -0.83 (0.97) | -1.87 (1.36) | -0.55 (0.76) | -1.59 (1.52) | -1.60 (1.57) | -0.53 (1.44) | -0.15 (0.44) | -0.30 (1.39) |
| Critical | 0.99 (2.37) | 0.11 (2.00) | 0.88 (1.82) | -1.33 (2.35) | -0.29 (0.95) | -1.18 (1.32) | -1.58 (0.68) * | -0.22 (1.47) | 1.92 (1.52) | 0.46 (1.40) | -0.32 (0.42) | 0.87 (1.34) |
| Results-oriented | 0.29 (2.50) | 1.35 (2.09) | -1.06 (1.92) | -0.05 (2.47) | -0.57 (0.99) | -1.62 (1.38) | -0.28 (0.77) | 1.57 (1.55) | 0.85 (1.60) | 1.45 (1.46) | -0.14 (0.45) | 1.48 (1.41) |
| Strategic | 0.74 (2.33) | 0.76 (1.95) | -0.01 (1.79) | 1.28 (2.30) | -0.85 (0.92) | 0.38 (1.29) | -1.39 (0.68) | 0.70 (1.44) | 2.42 (1.49) | -1.65 (1.36) | -0.72 (0.40) | -0.87 (1.32) |
| Autonomous | 1.54 (2.66) | 0.39 (2.25) | 1.16 (2.05) | -0.99 (2.64) | -0.38 (1.06) | -0.30 (1.48) | 0.00 (0.83) | -0.85 (1.65) | 0.52 (1.71) | 0.29 (1.57) | 0.40 (0.48) | 0.01 (1.51) |

**Table B3**

*Overview of Predictors in Single Regression Models for the Link Between Personality and LIWC Categories of Verbal Interactions*

| Predictor | LIWC Category | | | | | | | | | |
| --- | --- | --- | --- | --- | --- | --- | --- | --- | --- | --- |
|  | Words per Sentence | Word Count | I-Pronoun | We-Pronoun | Negations | Interrogatives | Positive Emotion Words | Negative Emotion Words | Cognitive Process Words | Time |
| **Emotional Stability** | -0.17 (0.27) | 5.32 (18.13) | -0.06 (0.43) | -0.02 (0.31) | 0.51 (0.39) | 0.05 (0.23) | 0.06 (0.28) | 0.1 (0.13) | 1.07 (0.8) | 1.26 (0.55) * |
| Relaxed | 0.07 (0.17) | -0.75 (11.45) | -0.17 (0.28) | 0.2 (0.19) | -0.07 (0.25) | -0.02 (0.15) | 0.15 (0.18) | -0.05 (0.09) | 1.30 (0.51) * | 0.6 (0.35) |
| Optimistic | -0.15 (0.18) | 7.57 (11.89) | 0.05 (0.29) | 0 (0.2) | 0.05 (0.26) | 0.09 (0.15) | 0.15 (0.18) | 0.04 (0.09) | -0.08 (0.54) | 1.02 (0.36) ** |
| Stress-resistant | 0.03 (0.19) | 2.82 (12.42) | -0.04 (0.3) | -0.15 (0.21) | 0.42 (0.27) | 0.01 (0.16) | 0.03 (0.19) | 0.05 (0.09) | 1.26 (0.55) * | 0.55 (0.38) |
| Decisive | -0.31 (0.19) | -0.2 (13.38) | 0.09 (0.32) | -0.13 (0.23) | 0.66 (0.29) * | 0.02 (0.17) | -0.3 (0.2) | 0.17 (0.1) | -0.76 (0.59) | 0.05 (0.41) |
|  |  |  |  |  |  |  |  |  |  |  |
| **Extraversion** | -0.29 (0.18) | 6.77 (12.66) | -0.33 (0.3) | -0.02 (0.22) | 0.71 (0.27) * | 0.16 (0.16) | -0.09 (0.19) | 0.14 (0.09) | -0.55 (0.56) | 0.33 (0.39) |
| Leading | -0.16 (0.14) | 4.14 (9.18) | -0.13 (0.22) | 0.02 (0.16) | 0.44 (0.20) * | 0.05 (0.12) | -0.1 (0.14) | 0.08 (0.07) | -0.53 (0.41) | -0.09 (0.28) |
| Communicative | -0.2 (0.16) | 7.29 (10.51) | -0.4 (0.25) | -0.1 (0.18) | 0.39 (0.23) | 0.23 (0.13) | 0 (0.16) | 0.15 (0.08) | -0.21 (0.48) | 0.49 (0.32) |
| Persuasive | -0.14 (0.2) | 12.55 (12.97) | -0.31 (0.32) | 0.01 (0.22) | 0.89 (0.28) ** | 0.13 (0.17) | 0 (0.2) | 0.1 (0.1) | 0.3 (0.59) | 0.35 (0.41) |
| Motivating | -0.45 (0.18) * | -3.32 (12.92) | -0.23 (0.31) | 0.02 (0.22) | 0.61 (0.28) * | 0.11 (0.16) | -0.16 (0.2) | 0.11 (0.09) | -1.20 (0.57) * | 0.49 (0.39) |
|  |  |  |  |  |  |  |  |  |  |  |
| **Openness** | 0.19 (0.22) | 11.27 (14.87) | -0.52 (0.36) | -0.09 (0.25) | -0.05 (0.32) | -0.11 (0.19) | -0.03 (0.23) | -0.06 (0.11) | 1.62 (0.66) * | 1.31 (0.45) ** |
| Abstract | 0.12 (0.12) | 9.49 (8.2) | -0.18 (0.2) | 0.07 (0.14) | -0.01 (0.18) | -0.06 (0.11) | -0.02 (0.13) | -0.01 (0.06) | 1.16 (0.37) ** | 0.38 (0.26) |
| Innovative | 0 (0.2) | -1.7 (13.1) | -0.51 (0.31) | -0.1 (0.22) | 0.06 (0.28) | -0.02 (0.17) | -0.13 (0.2) | -0.01 (0.1) | 0.37 (0.58) | 1.02 (0.39) * |
| Change-oriented | 0.2 (0.18) | 5.41 (12.34) | -0.1 (0.29) | -0.08 (0.21) | -0.25 (0.27) | -0.21 (0.16) | 0.04 (0.19) | -0.06 (0.09) | 1 (0.54) | 1.08 (0.37) ** |
| Open-minded | 0.08 (0.23) | 9.1 (15.2) | -0.73 (0.36) | -0.35 (0.25) | 0.17 (0.33) | 0.11 (0.2) | 0.08 (0.23) | -0.11 (0.11) | 0.93 (0.68) | 1.17 (0.46) * |

**Table B3 (Continued)**

| Predictor | Words per Sentence | Word Count | I-Pronoun | We-Pronoun | Negations | Interrogatives | Positive Emotion Words | Negative Emotion Words | Cognitive Process Words | Time |
| --- | --- | --- | --- | --- | --- | --- | --- | --- | --- | --- |
| **Altruism** | -0.32 (0.25) | 13.16 (16.59) | -0.19 (0.4) | 0.32 (0.28) | 0.54 (0.36) | 0.12 (0.22) | -0.06 (0.26) | 0.24 (0.12) | -0.87 (0.75) | 0.11 (0.52) |
| People-oriented | -0.11 (0.2) | 27.14 (12.79) * | -0.2 (0.33) | 0.29 (0.23) | 0.24 (0.3) | 0 (0.18) | 0.16 (0.21) | 0.11 (0.1) | 0.09 (0.63) | 0.09 (0.43) |
| Cooperating | -0.32 (0.2) | -4.64 (13.84) | 0.19 (0.34) | 0.19 (0.24) | 0.35 (0.3) | 0.04 (0.18) | -0.22 (0.21) | 0.14 (0.1) | -0.64 (0.62) | 0.13 (0.43) |
| Helpful | -0.3 (0.25) | 2.09 (16.97) | 0.2 (0.41) | 0.11 (0.29) | 0.52 (0.37) | 0.07 (0.22) | 0.07 (0.26) | 0.27 (0.12) * | -1.32 (0.75) | -0.24 (0.52) |
| Socially Confident | -0.18 (0.17) | 7.54 (11.37) | -0.43 (0.27) | 0.22 (0.19) | 0.35 (0.25) | 0.15 (0.15) | -0.1 (0.17) | 0.16 (0.08) | -0.65 (0.51) | 0.16 (0.35) |
|  |  |  |  |  |  |  |  |  |  |  |
| **Conscien-tiousness** | 0.15 (0.25) | 4.31 (16.92) | 0.18 (0.41) | -0.24 (0.29) | -0.02 (0.37) | 0.3 (0.22) | -0.49 (0.26) | -0.06 (0.13) | -0.74 (0.75) | -0.51 (0.52) |
| Organised | -0.01 (0.16) | -3.77 (10.71) | 0.25 (0.26) | -0.11 (0.18) | -0.18 (0.23) | 0.08 (0.14) | -0.31 (0.16) | 0 (0.08) | -0.58 (0.48) | -0.39 (0.33) |
| Meticulous | 0.11 (0.17) | 8.09 (11) | 0.04 (0.27) | 0 (0.19) | -0.04 (0.24) | 0.23 (0.14) | -0.21 (0.17) | -0.03 (0.08) | -0.75 (0.49) | -0.3 (0.34) |
| Rational | 0.39 (0.2) | 18.19 (14.01) | -0.28 (0.35) | -0.35 (0.24) | 0.03 (0.31) | 0.1 (0.19) | -0.31 (0.22) | -0.19 (0.11) | 0.88 (0.64) | 0 (0.45) |
| Persevering | -0.25 (0.29) | -24.23 (19.24) | 0.56 (0.47) | -0.29 (0.33) | 0.57 (0.42) | 0.41 (0.25) | -0.41 (0.3) | 0.13 (0.14) | -1.34 (0.87) | -0.49 (0.6) |
|  |  |  |  |  |  |  |  |  |  |  |
| Compounds |  |  |  |  |  |  |  |  |  |  |
| Ambitious | 0.07 (0.24) | 13.43 (16.01) | -0.11 (0.39) | -0.27 (0.27) | -0.14 (0.35) | -0.29 (0.21) | -0.16 (0.25) | 0.06 (0.12) | -0.23 (0.73) | -0.27 (0.5) |
| Critical | 0.29 (0.23) | 29.28 (14.85) | -0.46 (0.37) | -0.36 (0.26) | 0.2 (0.34) | 0.22 (0.2) | 0.05 (0.24) | 0.01 (0.12) | 1.55 (0.69) * | 0.31 (0.48) |
| Results-oriented | 0.28 (0.24) | 48.40 (14.05) ** | -0.23 (0.4) | 0.01 (0.28) | 0.07 (0.36) | 0.1 (0.21) | 0.31 (0.25) | -0.09 (0.12) | 1.28 (0.73) | 0.77 (0.5) |
| Strategic | 0.32 (0.22) | 32.31 (14.25) * | -0.27 (0.37) | -0.48 (0.25) | 0.32 (0.33) | 0.13 (0.2) | 0.13 (0.23) | -0.03 (0.11) | 2.10 (0.67) ** | 0.64 (0.47) |
| Autonomous | 0.19 (0.26) | 22.21 (17.28) | -0.58 (0.42) | 0.03 (0.3) | 0.58 (0.38) | 0.23 (0.22) | -0.45 (0.27) | 0.07 (0.13) | 0.16 (0.79) | 0.04 (0.54) |
